# Physicians' working conditions and job satisfaction: does hospital ownership in Germany make a difference?

**DOI:** 10.1186/1472-6963-9-148

**Published:** 2009-08-13

**Authors:** Stefanie Mache, Karin Vitzthum, Albert Nienhaus, Burghard F Klapp, David A Groneberg

**Affiliations:** 1 Institute of Occupational Medicine, Charité - School of Medicine, Free University and Humboldt University, Thielallee 69-73, 14195 Berlin, Germany; 2 Department of Respiratory Medicine, Hannover Medical School, Carl-Neuberg-Straße 1, 30625 Hannover, Germany; 3 Institution for Statutory Accident Insurance and Prevention in the Health and Welfare Services, Department of Occupational Health Research, Pappelallee 35-37, 22089 Hamburg, Germany; 4 Department of Medicine/Psychosomatics, Charité - School of Medicine, Free University and Humboldt University, Luisenstrasse 13a, 10117 Berlin, Germany

## Abstract

**Background:**

A growing number of German hospitals have been privatized with the intention of increasing cost effectiveness and improving the quality of health care. Numerous studies investigated what possible qualitative and economic consequences these changes issues might have on patient care.

However, little is known about how this privatization trend relates to physicians' working conditions and job satisfaction. It was anticipated that different working conditions would be associated with different types of hospital ownership. To that end, this study's purpose is to compare how physicians, working for both public and privatized hospitals, rate their respective psychosocial working conditions and job satisfaction.

**Methods:**

The study was designed as a cross-sectional comparison using questionnaire data from 203 physicians working at German hospitals of different ownership types (private for-profit, public and private nonprofit).

**Results:**

The present study shows that several aspects of physicians' perceived working conditions differ significantly depending on hospital ownership. However, results also indicated that physicians' job satisfaction does not vary between different types of hospital ownership. Finally, it was demonstrated that job demands and resources are associated with job satisfaction, while type of ownership is not.

**Conclusion:**

This study represents one of a few studies that investigate the effect of hospital ownership on physicians work situation and demonstrated that the type of ownership is a potential factor accounting for differences in working conditions. The findings provide an informative basis to find solutions improving physicians' work at German hospitals.

## Background

In recent years, a general trend toward hospital privatization has been growing in Germany. Between 1991 and 2007 the number of private hospitals increased from 262 up to 620 [1]. Presently 2087 hospitals are registered in Germany: 30% private for-profit hospitals, 32% public hospitals, and 38% private nonprofit hospitals.

In general, privatization aspires to make hospitals more cost-effective and to augment financial growth [2]. Previous research results confirmed that the privatization of hospitals had an impact on quality of care, productivity as well as economic issues [3].

However, in the debate regarding the pros and cons of privatization, there is only limited research on what possible effects privatization could have on German physicians' psychosocial and organizational working conditions as well as on their job outcomes.

At present, the desire to seek employment within German hospitals is declining [4]. This trend toward emigration among medical professionals has caused a shortage of qualified physicians within the German health care system. Therefore, we sought to examine possible reasons for this emigration wave by increasing the present understanding of job satisfaction in German hospitals.

A lot of research studies have been published about physicians' and nurses' job satisfaction [5-9]. However, none of these studies focused on differences between physicians' working conditions and job satisfaction at nonprofit and for-profit, public and private hospitals.

The purpose of this study was to compare the respective working conditions and job satisfaction for physicians working at public hospitals with those working at nonprofit and for-profit private hospitals.

Further research about this issue may help policy-makers and hospital administrators understand sources of discontent and encourage talented physicians to work within the domestic job market by improving working conditions in German hospitals, which would lead to overall improvements in health services.

### Psychosocial working conditions

The job demand resource model (JD-R model) is used to characterize working conditions by two categories: job demands and job resources [10].

### Job demands

Job demands refer to organizational, physical, psychological or social characteristics of work environment, demanding one's time and cognitive or physical efforts [10]. Examples of job demands on physicians at work are heavy patient load, working under schedule pressure, and being emotionally involved in the job [11].

Productivity seemed to increase with privatization, which implies that job demands should also increase. Previous studies showed health care employees faced with significantly higher workloads and physical demands after hospital privatization [12-14].

#### Hypothesis 1

Physicians' perceived job demands differ significantly depending on hospital ownership.

### Job resources

The second dimension of the JD-R model includes job resources, which are defined as psychological, social and organizational characteristics of work that may positively influence an employee's well-being [10]. Previous research studies demonstrated that these resources compensate for deleterious effects of job demands and encourage employees development [10]. Examples of job resources are 1) job autonomy and participation, 2) social support, and 3) quality of leadership.

Previous studies have shown that new management strategies of private hospitals often include a high level of employee involvement in the workplace, speculating that physicians will become more dedicated and thereby more productive [15]. In general, private hospitals are often characterized by shorter decision making processes and flatter hierarchical structures [16], which may indicate that physicians exercise greater autonomy than physicians in public hospitals.

#### Hypothesis 2

Physicians' perceived job resources differ significantly depending on hospital ownership.

### Job satisfaction

Job satisfaction is a multidimensional parameter, consisting of intrinsic factors, i.e. decision autonomy and recognition, and extrinsic factors, i.e. wages and job security [17].

Previous study results demonstrated that in terms of both pay and benefits physicians at private, for-profit hospitals received better compensation for their work than physicians at public hospitals [18,19].

Physicians working at public hospitals also complained that it was insufficiently rewarding in that they received little recognition for good work from superiors [20]. Moreover, physicians rated contentment with the process of performance assessment and positive working-relationships with supervisors higher in private organizations [21].

#### Hypothesis 3

Physicians' job satisfaction differs significantly depending on hospital ownership.

Previous research on nursing showed that job demands as well as job resources seemed to be significant factors for employees' job satisfaction [22,23]. These studies pointed out that job demands can reduce job satisfaction while job resources (e.g., support by colleagues) can increase job satisfaction. Since there are key differences between job demands of nurses and physicians, one cannot assume that these results are applicable to physicians.

Therefore the following hypotheses need to be investigated:

#### Hypothesis 4a

High levels of perceived job demands are associated with low levels of physicians' job satisfaction.

#### Hypothesis 4b

High levels of perceived job resources are associated with high levels of physicians' job satisfaction.

#### Hypothesis 4c

Levels of job demands and job resources help to explain the variance of physicians' job satisfaction.

### Personal resources

In addition, studies on the job demand-resource model have been restricted to external factors of job satisfaction. Consequently, the impact of employees' internal resources has been ignored, such as coping and adaptation skills for dealing with work demands [24]. Personal resources are features of one's personality that are associated with one's resiliency and individual sense of being able to successfully control and impact one's environment [24].

Since study results showed correlations between personal resources and job satisfaction [25,26], it can be expected that self-efficacious and optimistic physicians focus more on job resources than on job demands, and thus might be expected to report lower levels of job strain and higher levels of job satisfaction.

## Methods

### Study design and participants

The study was conducted as a cross-sectional survey using a standardized questionnaire to assess physicians' socio-demographic data, psychosocial working conditions and job satisfaction.

Participating hospitals, all located in a large German city, were chosen with regard to their specific care profile and type of ownership (private for-profit, private nonprofit, public nonprofit). All hospitals specialize in the same medical fields (internal medicine, surgery, pediatrics and neurology). They were almost equal in size; no significant differences were found between the hospitals in terms of the number of physicians, and nurses, patient beds or inpatient cases per year. During the data collection process, which took place between February and December 2008, one investigator conducted meetings informing the entire staff about the purpose of the study in order to encourage physicians to complete a questionnaire. They were requested to place it in a return box in their department. After three weeks, reminders were sent to the physicians by e-mail to increase the response rate. The total of 300 of questionnaires was distributed, of which 203 were returned (67% response rate).

#### Ethics

The ethical aspects were in full agreement with the Helsinki declaration. This study was approved by the Research Ethics Board at the University of Berlin.

### Questionnaire

#### Socio-demographic data of respondents

Items on socio-demographic data were included in the questionnaire; for example gender, and year of birth. In addition, details on physicians professional background were requested (i.e., area of specialty, time worked within the profession). Moreover, the type of hospital ownership at the institution the physician is working at was also assessed.

#### Psychosocial working factors and job satisfaction

The German version of the Copenhagen Psychosocial Questionnaire (COPSOQ) was used to assess job-related and psychosocial factors at work. The version used in this survey compromised 12 scales (Table 1) [27]. Various aspects of work, i.e., job demands (e.g. quantitative demands), job resources (i.e., influence at work, social support) as well as job outcomes (i.e., job satisfaction) are assessed by the COPSOQ.

**Table 1:** Dimensions of the COPSOQ, SWOP and BRCS questionnaires

**Scales**	**N of items**	**Items**
**Job demands**		
Quantitative demands	4	"Do you have to work very fast?"
Emotional demands	3	"Do you get emotionally involved in your work?"
Demands for hiding emotions	2	"Does your work require that you hide your feelings?"
**Job resources**		
Possibilities for development	4	"Do you have the possibility of learning new things through your work?"
Degree of freedom at work	4	"Can you decide when to take a break?"
Influence at work	4	"Do you have a large degree of influence concerning your work?"
Social relations	4	"Do you work isolated from your colleagues?"
Sense of community	3	"Is there a good co-operation between you and your colleagues?"
Social support	4	"How often do you get help and support from your colleagues?"
Quality of leadership	4	"To what extent would you say that your immediate superiors are good in solving conflicts?"
Feedback at work	2	"How often do you talk with your colleagues about how well you carry out your work?"
**Personal resources**		
Resilience	4	"Regardless of what happens to me, I believe I can control my reaction to it."
Self efficacy	5	"I face difficulties with relative ease because I can count on my abilities."
Optimism	2	"I'm always optimistic about my future."
Pessimism	2	"Things never go the way I want."
**Job outcome**		
Job satisfaction	4	"Regarding your work in general. How pleased are you with your job as a whole, everything taken into consideration?"

Reliability, validity and applicability of the COPSOQ were confirmed, scores ranging from satisfactory to good [28]. Cronbach's alpha coefficients for the items were higher than α = .61 (from α = .61 to α = .83) and all intercorrelations were measured between r = .30 - r = .68.

Previous studies corroborate good reliability [27]. Correlations between all the variables are illustrated in Additional file 1.

In general, Data were similar to the distribution among German hospital physicians reported by Nübling (n = 4254) [29].

#### Personal resources as covariates

Personal resources, such as resilience, self-efficacy, optimism and pessimism were integrated in the questionnaire (Table 1). The German version of the 'Brief Resilient Coping Scale' (BRCS) was included to ascertain abilities that help employees cope with stress in a highly adaptive way [30]. Additionally, the questionnaire 'Self-Efficacy, Optimism and Pessimism' (SWOP-K9) was also included [31]. The nine items in the SWOP-K9 may be summarized in three scales: self-efficacy, optimism and pessimism. The test quality criteria have been discussed elsewhere [31].

### Statistical data analysis

All categorical items relating to job demands, job resources and job satisfaction were transformed to a scale ranging from 0 (minimum value, e.g., "do not agree at all") to 100 points (maximum value, e.g., "fully agree") [27,32]. The category "does not apply" and item non-response were coded as missing values. Scale values were calculated as the mean of the values of the single items [32]. All scores were normally distributed.

Descriptive statistics and ANOVAs were performed to examine whether there were differences in working conditions as well as in job satisfaction between the three ownership types. Pearson product-moment correlation coefficients were used to measure correlations between the variables.

Hierarchical regression analysis was used to analyze the degree, to which job satisfaction was dependent on physicians' demands and resources. The variables were arranged in four hierarchical steps. In the first step, the background variables (gender, age and years of experience) were assessed. The second step contained the personal resources (i.e., optimism). The third step assessed the job demands (i.e., quantitative, emotional demands), while the final step contained the job resources (i.e., social support, leadership).

All p-values given were two-tailed. A p-value of less than .05 was considered significant. Values are given as mean and standard deviation (SD). Data were calculated using the SPSS^® ^software package for social sciences; Version 17.0.

## Results

### Demographic characteristics of participants

Socio-demographic data of the participating physicians working at hospitals of the three ownership types are illustrated in Table 2. Of the total number of participants in this study, 102 were female and 101 were male physicians with an average age of 34 years (SD = 6.54 years) and an average of 4 years (SD = 1.9 years) of working experience as a physician.

**Table 2:** Socio-demographic characteristics of the participating physicians, n = 203

	Absolute (n)	Relative (%)
**Hospital**		
Private for-profit	62	30.5
Public	77	38.0
Private nonprofit	64	31.5
		
**Age**		
Under 34 years	125	61.6
35-44 years	62	30.5
45-54 years	10	04.9
Over 55 years and older	5	02.5
		
**Gender**		
Male	101	49.8
Female	102	50.2
		
**Professional status**		
Intern/resident	167	82.8
Attending physician	25	12.3
Senior physician	8	3.9
Head of department/chief physician	3	1.5
		
**Area of specialization**		
Surgery	32	15.8
Gastroenterology	35	17.2
Cardiology	34	16.7
Hematology/Oncology	31	15.3
Pediatrics	39	19.2
Neurology	32	15.8
		
**Years of experience**		
Less than 1 year	24	11.8
1-2 years	24	11.8
3-5 years	74	36.5
More than 5 years	81	39.9

### Job demands and hospital ownership

The assumption of the first hypothesis conjectured that there is an influence on how physicians evaluate their job demands depending on the type of hospital ownership. Table 3 displays the mean values of job demands for each hospital and the ANOVA results.

**Table 3:** Psychosocial working conditions: means, univariate tests and covariate F-tests between the hospital types

	Private for-profit		Public		Private nonprofit		*F*-test	Covariates *F*-tests		
	M	SD	M	SD	M	SD	(d.f. = 2, 200)	(d.f. = 1, 202)		
								Age	Experience	Gender
								
**Job demands**										
Quantitative demands	61.59	14.31	67.17	13.73	67.46	11.83	4.24*	1.49	.63	.27
Emotional demands	59.81	14.19	59.22	14.60	62.37	16.65	.48	6.62**	.10	.01
Demands for hiding emotions	46.17	17.62	46.67	15.01	47.02	20.91	.07	1.96	.05	1.41
**Job resources**										
Possibilities for development	69.66	12.70	69.25	12.99	72.22	14.19	.81	1.84	.60	.16
Degree of freedom at work	49.76	16.26	51.33	14.72	49.14	15.48	.64	1.76	.01	.65
Influence at work	35.99	15.99	40.33	17.26	37.00	16.04	1.76	.26	.21	7.14**
Social relationships	59.68	19.79	55.67	20.68	52.98	19.41	1.76	4.31*	.21	3.11
Sense of community	74.33	13.35	72.33	12.64	79.37	12.33	5.58**	.26	.25	1.40
Social support	64.21	14.89	67.42	16.16	66.60	15.35	.91	.73	.02	.67
Quality of leadership	44.35	20.49	50.25	18.16	54.07	18.28	4.19*	1.04	4.50*	.60
Feedback at work	30.65	18.62	37.50	18.26	39.09	18.31	3.63*	.71	.45	.81
**Job outcome**										
Job satisfaction	57.72	15.21	58.93	11.78	61.22	12.36	1.32	.04	3.94*	2.28

* p < .05; ** p < .01.

Physicians in private for-profit hospitals perceived significantly fewer job demands than those in public and private nonprofit hospitals (F_2,200 _= 4.24, p = .01).

Emotional demands and demands on suppressing emotions were rated up to the same extent between all three forms of ownership.

### Job resources and hospital ownership

There were no significant results supporting that opportunities for career advancement, degrees of freedom at work and influence at work differ between the three ownership types (see Table 3).

Physicians working at private for-profit and nonprofit hospitals scored significantly higher for sense of community than did physicians at public hospitals (F_2,200 _= 5.58, p = .004). By contrast, social support was perceived higher at public and private nonprofit hospitals than at private for-profit hospitals; however, these differences did not reach significance. Quality of leadership was rated significantly lower in private for-profit hospitals than in public or private nonprofit hospitals (F_2,200 _= 4.19, p = .01). Receiving feedback on job performance also differed significantly (F_2,200 _= 3.63, p = .02).

To summarize, a portion of the evidence supports hypothesis 2: some job resources differ significantly between ownerships.

### Job satisfaction and hospital ownership

Table 3 compares the mean values of the responses of employees in public and private hospitals on job satisfaction. It revealed that job satisfaction tended to be highest at the private nonprofit hospitals. But the ANOVA-test indicated that the means did not differ significantly between the different hospital ownership types (F_2,200 _= 1.32, p = .26).

### Psychosocial working conditions, personal resources and job satisfaction

In Additional file 1, correlations between job satisfaction and job demands, job resources and personal resources are illustrated. In accordance with the corresponding hypotheses, this analysis has demonstrated negative correlations between job demands and job satisfaction (r = -.19 - r = -.42, p < .01). Strong positive correlations were found between job outcome and job resources (r = .24 - r = .52, p < .01). Positive correlations were also found between job satisfaction and self- efficacy (r = .35, p < .01) as well as between job satisfaction and optimism (r = .41, p < .01).

The regression model in Table 4 displays the ratios of variance and regression beta weights using job satisfaction as the dependent variable.

**Table 4:** Multiple hierarchical regressions (Ratios of variance and standardized beta weights of the last step in the regression)

**Predictors**	**Job satisfaction**		
	β	R^2^	ΔR^2^

**Socio-demographic variables**		.04	.04
Years of experience	-.14*		
Gender	-.03		
Age	-.02		
**Personal resources**		.24	.20
Resilience	.10		
Self-efficacy	.04		
Optimism	.12*		
Pessimism	.04		
**Job demands**		.37	.13
Quantitative demands	-.29***		
Emotional demands	-.02		
Demands for hiding emotions	.01		
**Job resources**		.59	.22
Possibilities for development	.21***		
Degree of freedom at work	.03		
Influence at work	.09		
Sense of community	.25***		
Social support	.04		
Social relations	-.03		
Quality of leadership	.16**		
Feedback at work	.05		

**Total R^2^**		.59	

* p < .05; ** p < .01; ***p < .001

Socio-demographic factors (age, gender and years of experience) accounted for a marginal portion of the variance (4%). Thereby, years of experience represented a significantly negative beta weight (β = -.14, p < .05). In the second step, personal resources accounted for an additional 20% of the variance. In the third step, job demands made up an additional 13% of the variance. Quantitative demands revealed a significantly negative beta weight (β = -.29, p < .001).

In the final step, included job resources accounted for an additional 22% of variance. Three job resources (possibilities for development, sense of community, and quality of leadership) revealed significantly positive beta weights (see Table 4).

In total, the model explained 59% of the observed variance in job satisfaction.

## Discussion

This is the first study to compare physicians working conditions and job satisfaction among private for-profit, public and private nonprofit hospitals. We collected survey data from 203 physicians and investigated whether levels of job demands and resources or job satisfaction differ between physicians working at hospitals with different ownership types. Furthermore, we examined the relationship between physicians' job demands and resources and their job satisfaction.

### Job demands

Regarding the first hypothesis, results indicate some differences in job demands between hospitals of different ownership. Quantitative job demands were higher scored at public hospitals than at private ones. This result is consistent with previous research [32,33].

Other studies have reported that public hospitals differ in patient turnover and organizational structures compared with private hospitals. However, physicians at public hospitals often treat patients with severe physical disorders who have been referred from smaller well-equipped or unspecialized hospitals. These multimorbid patients present challenges, which translate into higher working demands on physicians [34].

Regarding the emotional demands and suppression of emotions, no differences were found among the three groups, contrary to the postulation in hypothesis 1. Emotional demands may depend more on characteristics of a physician's daily work than on type of ownership, accounting for the lack of significance observed. Stressful emotional events (such as the treatment of dying people) occur at all hospitals, depending more likely on a physician's medical specialty than on type of ownership.

### Job resources

Comparisons of differences in job resources between the three groups of physicians showed significant variations in quality of leadership and feedback as well as sense of community, providing some support the second hypothesis.

Scores on quality of leadership and amount of regular feedback on work performance indicate that physicians at private nonprofit hospitals rated their supervisors and colleagues higher than physicians working at private for-profit hospitals.

As part of a goal-oriented job contract, physicians in private nonprofit hospitals often receive a financial bonus for excellent leadership [35]; this may motivate supervisors to pay more attention to issues concerning their colleagues [36,37].

In contrast, there were no significant differences in decision making processes, opportunities for career advancement and autonomy observed between the three ownership types. Since the study design focused mainly on junior physicians, it might be possible that occupational groups at different levels of hospital hierarchy receive different degrees of benefits [38].

In general, the findings demonstrate that individual opportunities to exert control over ones' work flow in medical care are relatively limited for all junior physicians and therefore do not depend on ownership.

It is reasonable to assume that junior physicians, who are still in training, have very limited autonomy over decision making and scheduling. In addition, a possible explanation for the low degree of autonomy, regardless of ownership, might be that in most cases the restraints placed on medical treatment and care by legal rules and regulations, leaving little room for individual decision making or self-determination [39].

For physicians working at private for-profit hospitals, perceptions of supportive job resources were comparatively lower than in the other two groups. Taking economic research into consideration, it seems that the open market economy, in which the private for-profit hospitals participate, creates a situation that limits some of the genuine human ideals and cooperative values, due to competition with other health care providers [40,41].

### Job satisfaction

No evidence was found to support the third hypothesis. Regarding mean values, job satisfaction was assessed to the same degree by all physicians and revealed mediocre scores. It was often demonstrated that private for-profit have superior management [42] and therefore it was assumed that job satisfaction should be rated higher. Alternatively, there is research suggesting that there are more similarities than differences regarding organizational aspects of different hospitals ownership types than previously expected [43]. Taking management strategies and German hospital structure into consideration, it is not surprising to find only moderate levels of job satisfaction among physicians. This finding could be associated with several factors, including the presence of extremely bureaucratic organizational structures as well as inefficient communication and cooperation among the personnel, which was expected to have negative consequences on attitudes of employees toward job satisfaction [32,44].

### Relationship between working conditions and job satisfaction

By conducting regression analyses, the relationship between job demands, job resources and personal resources and job satisfaction were examined. Our analysis suggests that these three features are almost equally important aspects of job satisfaction.

Job satisfaction may increase if physicians experienced more opportunities to advance their careers, team spirit, and better supervision. This finding supports previous results showing a similar relationship between job resources such as interpersonal relationships, cooperative arrangements and teamwork and higher job satisfaction [32,45].

Hence, these results have important implications for hospital management. Reducing sources of interpersonal conflict and promoting teamwork should rate high on the list of hospital managers' priorities.

Other variables included in this analysis are associated with specific dimensions of job satisfaction. For example, the pressure of quantitative demands and heavy workload reduce satisfaction significantly [46].

In summary, these results indicate that efforts to restructure hospitals, without regard to the effect on working conditions could potentially have dire consequences for physicians' job satisfaction, encouraging them to seek work elsewhere.

### Methodological considerations

A limitation of this study is its cross-sectional nature, which precludes causal analysis of the effect hospital ownership has on working conditions and job satisfaction. A longitudinal approach is needed fully examine the impact that privatization and for-profit business strategies have.

The second limitation refers to the complexity of variables that may have contributed to the results. Although the variables age, gender and years of experience have been controlled in the regression analysis, there are other factors and differences between hospital types that we have not included. Subsequent studies with larger samples should examine these and other factors in greater detail.

## Conclusion

In conclusion, the present study demonstrated that the type of ownership is a potential factor accounting for differences in working conditions. In contrast there was no significant variation in satisfaction levels between the three groups. This suggests that physician's satisfaction has less to do with hospital ownership type and more to do the general nature of the work [47]. The importance of the interplay between social demographic factors, psychosocial working conditions, and job satisfaction was demonstrated. However, based on the findings it is not possible to generally state that job demands are higher or job resources are better at nonprofit or for-profit hospitals.

Nevertheless, the results of this study provide an informative basis to develop solution-focused approaches improving physicians' work at German hospitals.

## Competing interests

The authors declare that they have no competing interests.

## Authors' contributions

SM conceived and designed the study. SM performed the investigation. SM analyzed the data. SM wrote the manuscript. SM, KV, AN, BFK and DAG interpreted the data and contributed substantially to its revision. All authors read and approved the final manuscript.

## Pre-publication history

The pre-publication history for this paper can be accessed here:



## Supplementary Material

Additional file 1 **Correlations between all included variables**. The data provided represent correlations between socio-demographic variables, personal resources, job demands, job resources and job satisfaction. Click here for file
