# Supplementary material for: Physicians' working conditions and job satisfaction: does hospital ownership in Germany make a difference?
Source: BMC Health Serv Res. 2009 Aug 13;9:148. doi: 10.1186/1472-6963-9-148 (PMC2735742; doi:10.1186/1472-6963-9-148)
Supplement: Additional file 1 — Correlations between all included variables. The data provided represent correlations between socio-demographic variables, personal resources, job demands, job resources and job satisfaction. [file 1472-6963-9-148-S1.doc]

**Additional file 1. Correlations between all included variables**

| **Variables** | 1 | 2 | 3 | 4 | 5 | 6 | 7 | 8 | 9 | 10 | 11 | 12 | 13 | 14 | 15 | 16 | 17 | 18 |
| --- | --- | --- | --- | --- | --- | --- | --- | --- | --- | --- | --- | --- | --- | --- | --- | --- | --- | --- |
| **Socio-demographic variables** |  |  |  |  |  |  |  |  |  |  |  |  |  |  |  |  |  |  |
| 1. Gender | - |  |  |  |  |  |  |  |  |  |  |  |  |  |  |  |  |  |
| 2. Age | -.16 | - |  |  |  |  |  |  |  |  |  |  |  |  |  |  |  |  |
| 3. Years of experience | -.09 | .61** | - |  |  |  |  |  |  |  |  |  |  |  |  |  |  |  |
| **Personal resources** |  |  |  |  |  |  |  |  |  |  |  |  |  |  |  |  |  |  |
| 4. Resilience | -,01 | .01 | -.06 | - |  |  |  |  |  |  |  |  |  |  |  |  |  |  |
| 5. Self-efficiency | -.21** | .00 | .04 | .33** | - |  |  |  |  |  |  |  |  |  |  |  |  |  |
| 6. Optimism | -.08 | .03 | -.03 | .30** | .52** | - |  |  |  |  |  |  |  |  |  |  |  |  |
| 7. Pessimism | .17* | .06 | .03 | -.27** | -.53** | -.34** | - |  |  |  |  |  |  |  |  |  |  |  |
| **Job demands** |  |  |  |  |  |  |  |  |  |  |  |  |  |  |  |  |  |  |
| 8. Quantitative demands | .04 | .13 | .08 | -.12 | -.05 | -.13 | .10 | - |  |  |  |  |  |  |  |  |  |  |
| 9. Emotional demands | -.02 | .24** | .15* | -.06 | -.13 | -.20** | .10 | .31** | - |  |  |  |  |  |  |  |  |  |
| 10. Demands for hiding emotions | .07 | .12 | .05 | -.23** | -.20** | -.19** | .30** | .26** | .23** | - |  |  |  |  |  |  |  |  |
| **Job resources** |  |  |  |  |  |  |  |  |  |  |  |  |  |  |  |  |  |  |
| 11. Influence at work | -.21** | .11 | .08 | .11 | .30** | .21** | -.22** | -.17* | -.05 | -.35** | - |  |  |  |  |  |  |  |
| 12. Degree of freedom at work | -.04 | -.11 | -.06 | .02 | .15* | .14* | -.16* | -.22** | -.12 | -.24** | .41** | - |  |  |  |  |  |  |
| 13. Possibilities for development | -.02 | .09 | .01 | .12 | .35** | .30** | -.16* | -.02 | .08 | -.16* | .35** | .20** | - |  |  |  |  |  |
| 14. Quality of leadership | -.02 | -.01 | -.13 | .16* | .18* | .22** | -.07 | -.23** | -.15* | -.21** | .32** | .16* | .32** | - |  |  |  |  |
| 15. Social support | -.03 | -.06 | -.03 | .11 | .19** | .19** | -.10 | -.08 | -.12 | -.18* | .25** | .21** | .29** | .54** | - |  |  |  |
| 16. Feedback at work | -.06 | .01 | -.05 | .02 | .13 | .10 | .01 | -.08 | -.04 | -.09 | .17* | -.04 | .16* | .39** | .35** | - |  |  |
| 17. Social relations | -.10 | -.15* | -.06 | .11 | .08 | .17* | -.10 | -.17* | -.11 | -.17* | .13 | .19** | .07 | .06 | .21** | .06 | - |  |
| 18. Sense of community | -.02 | .01 | .02 | .18* | .30** | .32** | -.25** | -.06 | -.02 | -.17* | .13 | .10 | .32** | .34** | .49** | .20** | .19** | - |
| **Job outcome** |  |  |  |  |  |  |  |  |  |  |  |  |  |  |  |  |  |  |
| 19. Job satisfaction | -.07 | -.10 | -.18** | .30** | .35** | .41** | -.21** | -.42** | -.19** | -.27** | .35** | .24** | .44** | .52** | .42** | .26** | .16* | .48** |

* p < .05; ** p < .01; -, not applicable
